# Racial Residential Segregation and Colorectal Cancer Mortality in the Mississippi Delta Region

**DOI:** 10.5888/pcd18.200483

**Published:** 2021-02-18

**Authors:** Aaron J. Kruse-Diehr, Justin T. McDaniel, Marquita W. Lewis-Thames, Aimee S. James, Musa Yahaya

**Affiliations:** 1 Department of Health, Behavior & Society, University of Kentucky College of Public Health, Lexington, Kentucky; 2 School of Human Sciences, Southern Illinois University, Carbondale, Illinois; 3 Department of Medical Social Science, Center for Community Health, Northwestern University Feinberg School of Medicine, Chicago, Illinois; 4 Department of Surgery, Division of Public Health Sciences, Washington University School of Medicine in St Louis, St Louis Missouri

## Abstract

**Introduction:**

Few studies have examined the effects of racial segregation on colorectal cancer (CRC) outcomes, and none has determined whether rurality moderates the effect of racial segregation on CRC mortality. We examined whether the effect of segregation on CRC mortality varied by rurality in the Mississippi Delta Region, an economically distressed and historically segregated region of the United States.

**Methods:**

We used data from the US Census Bureau and the 1999–2018 Surveillance, Epidemiology, and End Results (SEER) program to estimate mixed linear regression models in which CRC mortality rates among Black and White residents in Delta Region counties (N = 252) were stratified by rurality and regressed on White–Black residential segregation indices and 4 socioeconomic control variables.

**Results:**

Among Black residents, CRC mortality rates in urban counties were a function of a squared segregation term (b = 162.78, *P* = .01), indicating that the relationship between segregation and CRC mortality was U-shaped. Among White residents, main effects of annual household income (b = 29.01, *P* = .04) and educational attainment (b = 34.58, *P* = .03) were associated with CRC mortality rates in urban counties, whereas only annual household income (b = 19.44, *P* = .04) was associated with CRC mortality rates in rural counties. Racial segregation was not associated with CRC mortality rates among White residents.

**Conclusion:**

Our county-level analysis suggests that health outcomes related to racial segregation vary by racial, contextual, and community factors. Segregated rural Black communities may feature stronger social bonds among residents than urban communities, thus increasing interpersonal support for cancer prevention and control. Future research should explore the effect of individual-level factors on colorectal cancer mortality.

**Summary**

**What is already known on this topic?**

Colorectal cancer mortality rates are higher than the national average among rural residents of the Mississippi Delta Region. Little is known about the interaction between rurality and racial segregation.

**What is added by this report?**

We found that colorectal cancer mortality was higher among Black residents in urban Delta Region counties with low and high levels of racial segregation, but this relationship was less evident among Black residents in rural Delta Region counties.

**What are the implications for public health practice?**

Further research should be conducted in segregated rural communities to better understand protective factors for Black residents against colorectal cancer mortality. Practitioners should partner with existing organizations to leverage social networks when developing and implementing colorectal cancer interventions.

## Introduction

Colorectal cancer (CRC) is the third most commonly diagnosed cancer and the second leading cause of cancer-related deaths among adults in the United States (1). Although CRC mortality rates decreased from 28.6 per 100,000 population in 1976 to 14.1 in 2014, higher mortality rates persist in the lower Mississippi Delta Region, which includes parts of Arkansas, Tennessee, Louisiana, Mississippi, Kentucky, Missouri, and Illinois (2). Because colorectal cancer mortality was approximately 40% higher in the Delta Region than in the rest of the United States from 2009–2011, a cluster of 94 Delta counties has been designated as the nation’s largest hotspot for CRC mortality (2). Additionally, many of these counties have also been found to be hotspots for early-onset CRC (3). Compared with their non-Delta counterparts, Delta counties have a greater proportion of low-income Black residents, lower median household income, lower educational attainment, higher rates of obesity, less access to exercise opportunities, higher smoking rates, and a less nutritious food environment (4). These factors, among others, contribute to higher mortality in the Delta Region.

The largely rural Delta Region is heavily segregated by race, with poorer health outcomes concentrated in census blocks with predominantly Black residents (5), and although urban residential segregation has decreased in the United States, the opposite trend has occurred in rural areas (6). Residential segregation has been linked to poorer quality education, reduced access to employment, more concentrated poverty, higher infant mortality rates, and reduced access to both primary and specialty health care, among other negative outcomes (7). These disparities may be even more pronounced in segregated rural areas, where factors such as poverty and travel distance make it difficult to access resources.

Although the association between rurality and increased cancer mortality is clear (8), the confounding effect of race and residential segregation is blurry. A systematic review of segregation and racial cancer disparities noted that 70% of included studies found that segregation contributed in some way to cancer mortality, though not always negatively (9). In highly segregated areas, studies reported lower breast cancer mortality among Black women but not White women (10), higher breast cancer mortality among Black women but not White women (11), and no associations between segregation and breast cancer mortality among Black women (12). For lung cancer, segregation has been linked to higher mortality rates among Black residents, but among White residents living in segregated areas, the association between lung cancer mortality rates is either weaker (13) or nonexistent (14). Further complicating the interpretation of these associations, studies routinely operationalize racial segregation in multiple ways — ranging from the percentage of Black people living in a given area (9) to measuring dissimilarity (ie, unevenness and clustering) of racial distribution (13) — and at different levels, including the census block group (10,13) and the metropolitan/micropolitan statistical area (11). Given that evidence of the effect of racial segregation on cancer outcomes is inconclusive, additional investigation is needed to better understand these associations to assess allocation of resources and education for underserved and disparate populations in racially segregated areas.

For Delta Region residents, accounting for racial residential segregation is an important, but less investigated, structural and social determinant of health (5,15). Previous studies investigated relationships between segregation and CRC outcomes throughout the continuum, including early-stage CRC diagnosis (16), late-stage CRC diagnosis (17), and treatment (15). To date, few studies have examined the effects of racial segregation on CRC outcomes, and none has determined if the effect of racial segregation on CRC mortality among Black and White residents varies by rurality. Given that the Delta Region 1) encompasses the largest hotspot for CRC mortality, 2) comprises both rural and urban counties (as classified by rural–urban continuum codes), and 3) includes regions that have been historically racially segregated, it provides a unique context within which to achieve the objective of this study: to describe relationships between racial residential segregation and CRC mortality and determine whether effects of segregation differ by race and between rural and urban Delta Region residents.

## Methods

### Study design and outcome variable

We used an ecologic study design, with counties in the Delta Region as the unit of analysis (N = 252), to determine whether the relationship between racial residential segregation and CRC mortality rates — our main outcome variable — among Black and White Delta residents varied by rurality. We calculated age-standardized CRC mortality rates per 100,000 for White and Black residents separately in each Delta county for the period 1999–2018 using the National Cancer Institute’s Surveillance, Epidemiology, and End Results (SEER) SEER*Stat (version 8.3.5) software, which collects data from both SEER cancer registries and the National Center for Health Statistics (18).

### Independent variables

We measured racial residential segregation for White and Black residents in each Delta county by using the multilevel index of dissimilarity (MLID), which measures the spatial clustering of segregation (19). We calculated the MLID for each Delta county using population count data from the 2011–2015 US Census Bureau for White and Black residents in 3 nested within-county census geographies: block groups, tracts, and county subdivisions (20). We used the Missouri Census Data Center Geographic Correspondence Engine (Geocorr) to map tracts onto county subdivisions, because some census tracts overlapped county subdivision boundaries (21). The MLID can range from 0 (no segregation) to 1 (total segregation) (19). We calculated the MLID for each county by using the MLID package in RStudio version 3.6.1.

We determined county rurality using 2013 rural–urban continuum codes (RUCCs) from the US Department of Agriculture (22). RUCCs range from 1 (counties in metropolitan areas with populations >1,000,000) to 9 (completely rural or an urban population <2,500, not adjacent to a metropolitan area). Similar to the approach used by Zahnd and colleagues (23), we dichotomized all RUCCs to indicate whether a county was urban (RUCCs 1 to 3) or rural (RUCCs 4 to 9).

### Control variables

We included several control variables in our analysis to isolate the effects of rurality and racial residential segregation on CRC mortality rates. Manser and Bauerfeind’s (24) systematic review indicated that CRC mortality was strongly associated with socioeconomic factors, such as low income, low levels of education, and overcrowding. We included these factors as direct measures of socioeconomic status.

First, using the same data we used to calculate county MLIDs, we calculated the proportion of each county’s population that was Black and the proportion of each county’s population that was White. Second, we determined the proportion of Black and White residents, separately, in each county, who reported an annual household income of less than $20,000 using 2011–2015 data from the US Census Bureau (20). Third, we determined the proportion of Black and White residents, separately, in each county, who reported having never completed high school using 2011–2015 data from the US Census Bureau (20). Fourth, we determined the proportion of Black and White residents, separately, in each county, who reported living arrangements with more than 1 occupant per room in the house (ie, overcrowding) using 2011–2015 data from the US Census Bureau (20).

### Data analysis

Because counties with fewer than 10 deaths caused by CRC were suppressed to ensure confidentiality, we analyzed 169 Delta Region counties with data on CRC mortality rates among Black residents and 248 counties with data on CRC mortality rates among White residents. That is, we conducted complete case analysis because the data missing were not missing at random; data on CRC mortality among Black residents had 67% missingness, which is 27 percentage points greater than current guidance for the use of imputation (25). We estimated 4 mixed linear regression models to address our research questions. In the first and second models, which were stratified by rural or urban status, we regressed CRC mortality rates among Black residents on MLIDs while controlling for county-level socioeconomic factors among Black residents. These models also included a quadratic term for MLID. In the third and fourth models, which were also stratified by rural and urban status, we regressed CRC mortality rates among White residents on MLIDs while controlling for county-level socioeconomic factors among White residents. These models included a quadratic term for MLID as well. All 4 models included a random intercept for the nesting of counties within states. Because we obtained all data from de-identified public use data sets, institutional review board approval was not required for this study. We used Stata version 16 (StataCorp LLC) to estimate models, and all figures were produced using ESRI ArcGIS version 10.5.1.

## Results

**Figure 1:**
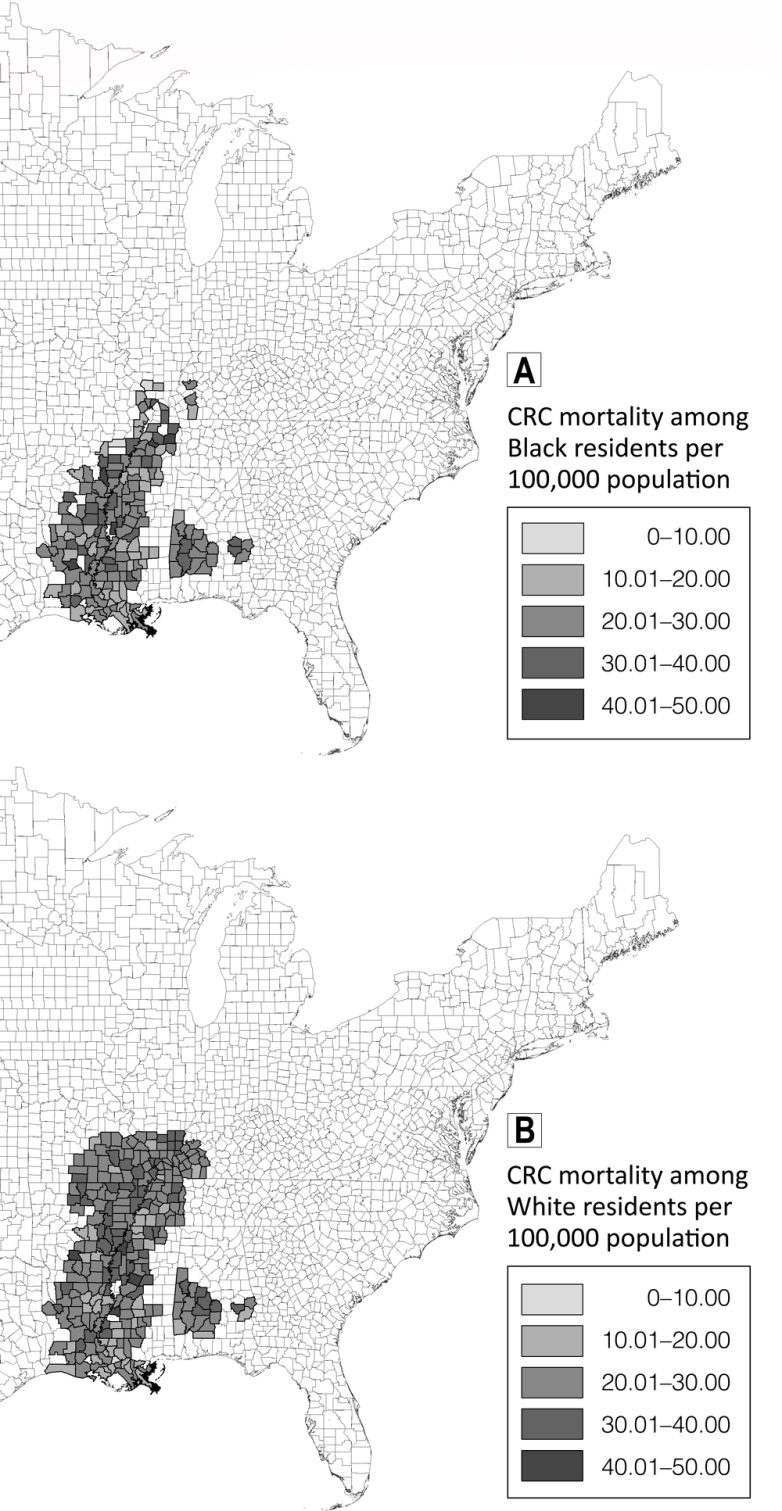
Colorectal cancer mortality rates per 100,000 population among A, Black residents and B, White residents in counties in the Mississippi Delta Region, 1999–2018. Map created using ESRI ArcGIS version 10.5.1. Abbreviation: CRC, colorectal cancer.

Although county-level CRC mortality rates were higher on average among White residents than among Black residents (Table 1), we observed greater variability in rates among Black residents. For example, we observed the highest (45.77 per 100,000) and the lowest (7.54 per 100,000) CRC mortality rates in urban counties among Black residents. CRC mortality rates among Black residents were highest in Crockett County, Tennessee (45.77 per 100,000), and Sharkey County, Mississippi (44.18 per 100,000), whereas CRC mortality rates among White residents were highest in Holmes County, Mississippi (44.80 per 100,000), and Dallas County, Arkansas (42.35 per 100,000) (Figure 1).

**Table 1:** Average Colorectal Cancer Mortality Rates and Aggregate Sociodemographic Characteristics for Mississippi Delta Region Counties, by Rural–Urban^a^ Designation and Race, 1999–2018

Variable	Mean (SD)			
	Black Residents		White Residents	
	Rural	Urban	Rural	Urban
County population with <$20,000 in annual household income, %^b^	42.4 (21.7)	37.6 (10.4)	24.4 (4.5)	18.5 (4.9)
County population with <high school education, %^b^	28.8 (14.3)	23.5 (7.0)	17.6 (4.2)	13.9 (4.6)
County population living in a residence with >1 occupant per room, %^b^	2.9 (3.1)	4.6 (3.2)	1.7 (1.1)	1.8 (1.0)
County population that is Black or White, %^b^	27.5 (24.7)	32.0 (19.2)	72.5 (24.7)	68.0 (19.2)
County-level residential racial segregation^b^^,^ c	0.5 (0.2)	0.5 (0.1)	—	—
Colorectal cancer mortality per 100,000^d^	25.6 (6.2)	21.0 (7.0)	27.5 (6.1)	21.7 (5.3)

a Determined by using 2013 rural-urban continuum codes (RUCCs) from the US Department of Agriculture (22). RUCCs range from 1 (counties in metropolitan areas with populations >1,000,000) to 9 (ie, completely rural or an urban population >2,500, not adjacent to a metropolitan area). All RUCCs dichotomized to indicate urban (RUCCs 1 to 3) or rural (RUCCs 4 to 9).

b Data source: US Census Bureau (20).

c Measured by the multilevel index of dissimilarity, which measures the spatial clustering of segregation in a county and is not specific to 1 racial group; it can range from 0 (no segregation) to 1 (total segregation) (19).

d Data source: National Cancer Institute (18).

**Figure 2:**
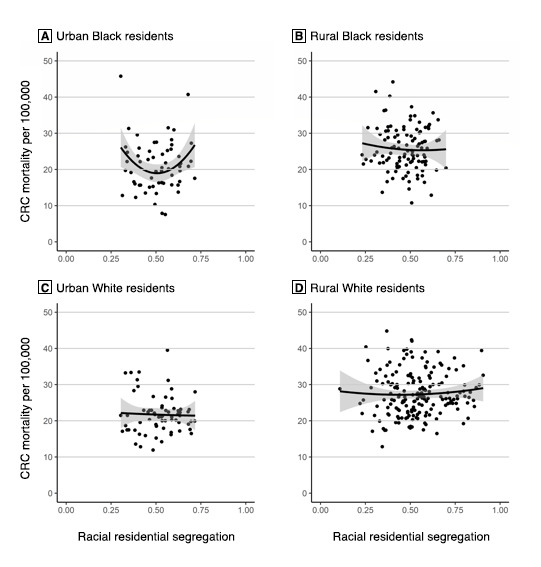
The effects of county urbanity and rurality on the relationship between Black–White residential segregation, as measured by the multilevel index of dissimilarity (MLID), which measures the spatial clustering of segregation (19), and colorectal cancer mortality rates among Black and White residents in Mississippi Delta region counties. A, Urban Black residents; B, Rural Black residents; C, Urban White residents; D, Rural White residents. Shading indicates 95% CIs.

The mixed linear regression model for CRC mortality among Black residents in rural counties (χ^2^ = 6.2, *P* = .40) showed that racial segregation — including a quadratic form of segregation (ie, a U-shaped relationship) — was not significantly associated with CRC mortality (Table 2). However, the model for CRC mortality among Black residents in urban counties (χ^2^ = 17.6, *P* = .008) showed that a quadratic term for racial segregation was significantly associated with CRC mortality (b = 162.78, *P* = .01). As such, starting with counties that had an MLID of 0, the slope is such that CRC mortality among Black residents would decrease by 158.99 per 100,000 for each additional unit of the MLID — that is, if the slope remained unchanged; however, our model discredits the idea of an unchanged slope. Each unit added to the MLID increased the slope of the CRC mortality rate among Black residents by 162.78 per 100,000. In this model, the coefficient of the square term was positive, indicating that the relationship between the MLID and CRC mortality among Black residents was convex (Figure 2).

**Table 2:** Factors Associated With Colorectal Cancer Mortality Rates Among Black Residents and White Residents in Counties in the Mississippi Delta Region, 1999–2018^a^

Variable	Models for Black Residents				Models for White Residents			
	Rural		Urban		Rural		Urban	
	b (SE)	*P* Value	b (SE)	*P* Value	b (SE)	*P* Value	b (SE)	*P* Value
Fixed intercept	24.84 (11.31)	.03	50.35 (17.05)	<.001	31.43 (4.64)	<.001	12.18 (10.77)	.26
Proportion of racial group in a county with <$20,000 in annual household income^a^	5.39 (9.09)	.55	−3.52 (10.83)	.75	19.44 (9.39)	.04	29.01 (13.90)	.04
Proportion of racial group in a county with <high school education^a^	−0.90 (9.41)	.92	46.88 (15.66)	<.01	8.96 (9.88)	.36	34.58 (15.92)	.03
Proportion of racial group living in a residence with >1 occupant per room (ie, overcrowding)^a^	−41.29 (26.74)	.12	−9.69 (36.77)	.79	−47.75 (35.33)	.18	14.03 (56.31)	.80
Population proportion^b^	5.78 (3.22)	.07	−2.66 (4.72)	.57	−19.43 (2.49)	<.001	−12.29 (3.06)	<.001
Residential segregation^a^^,^ c	−10.97 (43.27)	.80	−158.99 (66.82)	.02	8.05 (13.42)	.55	23.81 (41.71)	.57
Segregation × segregation	12.47 (46.62)	.79	162.78 (64.83)	.01	1.96 (12.06)	.87	−14.61 (39.99)	.72
Random intercept	3.65 (4.09)	—	5.46 (6.90)	—	11.78 (7.01)	—	2.73 (3.08)	—

a Data source: US Census Bureau (20).

b Black population proportion for the Black model; White population proportion for the White model.

c Measured by the multilevel index of dissimilarity, which measures the spatial clustering of segregation in a county and is not specific to 1 racial group; it can range from 0 (no segregation) to 1 (total segregation) (19).

The model for CRC mortality among White residents in rural counties (χ^2^ = 72.2, *P* < .001) showed that racial segregation — including a quadratic form of segregation — was not significantly associated with CRC mortality. The model for CRC mortality among White residents in urban counties (χ^2^ = 36.1, *P* < .001) also showed that racial segregation was not significantly associated with CRC mortality; however, the models for CRC mortality among White residents showed that educational attainment and annual income were more important than racial segregation as predictors of CRC mortality. Specifically, in rural counties, a 1 percentage-point increase in the county percentage of White residents with less than $20,000 in annual household income was associated with an increase of 19.44 per 100,000 in CRC mortality. In urban counties, a 1 percentage-point increase in the county percentage of White residents with less than $20,000 in annual household income was associated with an increase of 29.01 per 100,000 in CRC mortality. Additionally, in urban counties, a 1 percentage-point increase in the county percentage of White residents with less than a high school education was associated with an increase of 34.58 per 100,000 in CRC mortality.

## Discussion

Our models suggested that urban Delta Region counties with low and high, but not moderate, levels of racial segregation had higher CRC mortality rates among Black residents, a finding aligned with other research on CRC disparities (15,26). However, this relationship was less evident in rural Delta Region counties. Although this finding may seem surprising, the relationship between residential segregation and cancer outcomes among Black people remains unclear. Some studies found poor cancer outcomes (as we did for urban counties) (13,14), while others showed protective effects (10,17) and others reported no association (11,12,27). Our discovery that moderately segregated urban Delta Region counties had lower CRC mortality among Black residents than counties with low or high levels of segregation is, to our knowledge, a novel finding. Clearly, the pathways by which the interaction of urbanity/rurality and race affect CRC outcomes deserves additional exploration in health research, particularly given varying levels of county-level segregation. A few hypotheses might provide insight into our novel findings.

One potential explanation for our findings is that segregated rural communities may have unique features that do not exist in their segregated rural analogs. Racial or ethnic enclaves — geographic areas marked by large concentrations of people of similar races or ethnicities that often feature organizations led by members of these communities — have been shown to impart health benefits via different pathways, such as smaller and more racially concordant social networks (10), increased social capital and support (9,10), and less exposure to racism-related stress (27). However, other highly segregated areas may be cut off from resources, access, and knowledge (7), thus perpetuating unequal balances of power or resources and leaving communities of color with smaller social networks and less support (10). These disparities may be more pronounced in urban areas and social bonds may be stronger in segregated rural communities, thus contributing to urban–rural differences in cancer outcomes. Perhaps, too, social bonds in Black rural communities yield stronger or more effective interpersonal support to promote screening for preventable cancers such as colorectal cancer, a hypothesis echoed by Moss and colleagues (28), who found higher CRC screening rates in highly segregated counties than in those with less segregation.

Despite these possible differences, it is critical to remember that racial residential segregation is a system of oppression that comprises multiple factors that affect long-term health outcomes. Because of segregation, Black communities have historically entrenched and socially and politically enforced barriers to economic, educational, and health resources, implications of which continue to be felt today. Although our study identified a stronger relationship between CRC mortality and Black segregation in urban Delta Region counties than in rural Delta Region counties, it is important to acknowledge that multiple factors likely drive this relationship, thus underscoring the necessity for continued research dedicated to understanding the long-term effects of segregation on health outcomes and how these effects might produce different outcomes in both urban and rural residents in different geographic regions of the country.

Our county-level data do not fully capture data on individual-level factors — such as comorbidities, screening data, median age of death, or other risk factors — that might partly explain our findings. Data from the 2012–2015 Behavioral Risk Factor Surveillance System show that rural Black residents self-report lower levels of health-related quality of life, higher cost-related barriers to seeking treatment, lower CRC screening rates, and more comorbidities than rural White residents (29). Furthermore, precancerous polyps, many of which have little or no symptoms, can take upwards of a decade to progress to CRC (30). Perhaps, then, rural Black residents in the Delta Region are dying prematurely from complications of other causes (ie, multiple chronic conditions) *before* dying from the slower developing consequences of CRC. Given that the Delta Region as a whole has one of the lowest life expectancies in the country (4), our findings might not fully show the entire picture on trends in CRC mortality among Black people in the Delta Region.

Our study has several limitations. First, we examined only 1 geographic area of the United States, the Mississippi Delta Region. Although we selected this region purposely because of its large burden of CRC mortality (2), researchers should investigate other rural areas to determine whether they differ from the Delta. Second, data on CRC mortality among Black residents were suppressed in many counties. The averages computed in our study may not reflect the region as a whole.

Third, our study was limited by the snapshot of health represented from 1999–2018, and general implications about the effects of a socially and legislatively enforced historical phenomenon like segregation on health outcomes. Fourth, given that the county was the unit of analysis in this study, we were unable to control for individual-level covariates (eg, stage at diagnosis, median age, comorbidity scores, and individual insurance coverage) that may have partly explained our findings. Fifth, no publicly available data set breaks down CRC screening at the county level by race; it is possible that screening differences account for a proportion of our findings. Finally, our study is correlational in nature and, as such, no causal effects can be inferred based on our findings.

To date, few studies have examined the effects of racial residential segregation on CRC outcomes, and to the best of our knowledge, none has determined whether the effect of segregation on CRC mortality among Black and White residents varies by rurality. Here, we used the Mississippi Delta Region as a frame of reference, given its history of racial segregation, combination of rural and urban counties, and highest incidence of CRC mortality of any hotspot in the United States (2). We found that urban counties with low and high levels of racial segregation had higher rates of CRC mortality among Black residents than moderately segregated urban counties. This relationship was less pronounced in rural counties. Furthermore, segregation was not a significant factor in CRC mortality among White residents in urban or rural counties. Our findings suggest that segregation affects White and Black residents differently, especially in rural areas. Future research should examine individual-level factors that may help explain this rural–urban disparity. Collectively, these findings can help inform community-engaged evidence-based practices to reduce CRC cancer burden in rural segregated areas.

## References

[1] American Cancer Society. Cancer facts & figures 2019. Atlanta (GA): American Cancer Society; 2019.

[2] Siegel RL , Sahar L , Robbins A , Jemal A . Where can colorectal cancer screening interventions have the most impact? Cancer Epidemiol Biomarkers Prev 2015;24(8):1151–6. 10.1158/1055-9965.EPI-15-0082 26156973

[3] Rogers CR , Moore JX , Qeadan F , Gu LY , Huntington MS , Holowatyj AN . Examining factors underlying geographic disparities in early-onset colorectal cancer survival among men in the United States. Am J Cancer Res 2020;10(5):1592–607. 32509399 PMC7269786

[4] Gennuso KP , Jovaag A , Catlin BB , Rodock M , Park H . Assessment of factors contributing to health outcomes in the eight states of the Mississippi Delta Region. Prev Chronic Dis 2016;13:E33. 10.5888/pcd13.150440 26940300 PMC4778371

[5] Harvey MH . Consensus-based community development, concentrated rural poverty, and local institutional structures: the obstacle of race in the lower Mississippi Delta. Community Dev (Columb) 2013;44(2):257–73. 10.1080/15575330.2012.734840

[6] Parisi D , Lichter DT , Taquino MC . Multi-scale residential segregation: black exceptionalism and America’s changing color line. Soc Forces 2011;89(3):829–52. 10.1353/sof.2011.0013

[7] Williams DR , Lawrence JA , Davis BA . Racism and health: evidence and needed research. Annu Rev Public Health 2019;40(1):105–25. 10.1146/annurev-publhealth-040218-043750 30601726 PMC6532402

[8] Henley SJ , Anderson RN , Thomas CC , Massetti GM , Peaker B , Richardson LC . Invasive cancer incidence, 2004-2013, and deaths, 2006-2015, in nonmetropolitan and metropolitan counties — United States. MMWR Surveill Summ 2017;66(14):1–13. 10.15585/mmwr.ss6614a1 28683054 PMC5879727

[9] Landrine H , Corral I , Lee JGL , Efird JT , Hall MB , Bess JJ . Residential segregation and racial cancer disparities: a systematic review. J Racial Ethn Health Disparities 2017;4(6):1195–205. 10.1007/s40615-016-0326-9 28039602

[10] Warner ET , Gomez SL . Impact of neighborhood racial composition and metropolitan residential segregation on disparities in breast cancer stage at diagnosis and survival between black and white women in California. J Community Health 2010;35(4):398–408. 10.1007/s10900-010-9265-2 20358266 PMC2906635

[11] Russell EF , Kramer MR , Cooper HLF , Gabram-Mendola S , Senior-Crosby D , Jacob Arriola KR . Metropolitan area racial residential segregation, neighborhood racial composition, and breast cancer mortality. Cancer Causes Control 2012;23(9):1519–27. 10.1007/s10552-012-0029-4 22825071

[12] Pruitt SL , Lee SJ , Tiro JA , Xuan L , Ruiz JM , Inrig S . Residential racial segregation and mortality among black, white, and Hispanic urban breast cancer patients in Texas, 1995 to 2009. Cancer 2015;121(11):1845–55. 10.1002/cncr.29282 25678448 PMC5308210

[13] Hayanga AJ , Zeliadt SB , Backhus LM . Residential segregation and lung cancer mortality in the United States. JAMA Surg 2013;148(1):37–42. 10.1001/jamasurgery.2013.408 23324839

[14] Johnson AM , Johnson A , Hines RB , Bayakly R . The effects of residential segregation and neighborhood characteristics on surgery and survival in patients with early-stage non-small cell lung cancer. Cancer Epidemiol Biomarkers Prev 2016;25(5):750–8. 10.1158/1055-9965.EPI-15-1126 27197137

[15] Hao Y , Landrine H , Jemal A , Ward KC , Bayakly AR , Young JL Jr , Race, neighbourhood characteristics and disparities in chemotherapy for colorectal cancer. J Epidemiol Community Health 2011;65(3):211–7. 10.1136/jech.2009.096008 19959651

[16] Haas JS , Earle CC , Orav JE , Brawarsky P , Neville BA , Williams DR . Racial segregation and disparities in cancer stage for seniors. J Gen Intern Med 2008;23(5):699–705. 10.1007/s11606-008-0545-9 18338215 PMC2324162

[17] Mobley LR , Scott L , Rutherford Y , Kuo T-M . Using residential segregation to predict colorectal cancer stage at diagnosis: two different approaches. Ann Epidemiol 2017;27(1):10–9. 10.1016/j.annepidem.2016.11.008 27939165 PMC5272810

[18] National Cancer Institute. SEER*Stat software, version 8.3.5. Bethesda, MD: National Cancer Institute; 2018.

[19] Harris R , Owen D . Implementing a multilevel index of dissimilarity in R with a case study of the changing scales of residential ethnic segregation in England and Wales. Environ Plan B Urban Anal City Sci. 2017;45(6):1003–21. 10.1177/2399808317748328

[20] US Census Bureau. Explore Census data. https://data.census.gov/cedsci. Accessed February 11, 2019.

[21] Missouri Census Data Center. Geocorr 2014: geographic correspondence engine. 2016. http://mcdc.missouri.edu/applications/geocorr2014.html. Accessed February 11, 2019.

[22] US Department of Agriculture. Rural–urban continuum codes. 2013. https://www.ers.usda.gov/data-products/rural-urban-continuum-codes. Accessed February 2, 2019.

[23] Zahnd WE , James AS , Jenkins WD , Izadi SR , Fogleman AJ , Steward DE , Rural–urban differences in cancer incidence and trends in the United States. Cancer Epidemiol Biomarkers Prev 2018;27(11):1265–74. 10.1158/1055-9965.EPI-17-0430 28751476 PMC5787045

[24] Manser CN , Bauerfeind P . Impact of socioeconomic status on incidence, mortality, and survival of colorectal cancer patients: a systematic review. Gastrointest Endosc 2014;80(1):42–60.e9. 10.1016/j.gie.2014.03.011 24950641

[25] Jakobsen JC , Gluud C , Wetterslev J , Winkel P . When and how should multiple imputation be used for handling missing data in randomised clinical trials — a practical guide with flowcharts. BMC Med Res Methodol 2017;17(1):162. 10.1186/s12874-017-0442-1 29207961 PMC5717805

[26] Coughlin SS , Richards TB , Thompson T , Miller BA , VanEenwyk J , Goodman MT , Rural/nonrural differences in colorectal cancer incidence in the United States, 1998–2001. Cancer 2006;107(5 Suppl):1181–8. 10.1002/cncr.22015 16802323

[27] Mobley LR , Kuo T-M , Scott L , Rutherford Y , Bose S . Modeling geospatial patterns of late-stage diagnosis of breast cancer in the US. Int J Environ Res Public Health 2017;14(5):484. 10.3390/ijerph14050484 28475134 PMC5451935

[28] Moss JL , Ehrenkranz R , Perez LG , Hair BY , Julian AK . Geographic disparities in cancer screening and fatalism among a nationally representative sample of US adults. J Epidemiol Community Health 2019;73(12):1128–35. 10.1136/jech-2019-212425 31615890

[29] James CV , Moonesinghe R , Wilson-Frederick SM , Hall JE , Penman-Aguilar A , Bouye K . Racial/ethnic health disparities among rural adults — United States, 2012–2015. MMWR Surveill Summ 2017;66(23):1–9. 10.15585/mmwr.ss6623a1 29145359 PMC5829953

[30] Simon K . Colorectal cancer development and advances in screening. Clin Interv Aging 2016;11:967–76. 10.2147/CIA.S109285 27486317 PMC4958365

